# Nonlinear diversification rates of linguistic phylogenies over the Holocene

**DOI:** 10.1371/journal.pone.0213126

**Published:** 2019-07-17

**Authors:** Marcus J. Hamilton, Robert S. Walker

**Affiliations:** 1 Department of Anthropology, University of Texas at San Antonio, San Antonio, TX, United States of America; 2 Santa Fe Institute, Santa Fe, New Mexico, NM, United States of America; 3 Department of Anthropology, University of Missouri, Columbia, MO, United States of America; University of Toronto, CANADA

## Abstract

The expansion of the human species out of Africa in the Pleistocene, and the subsequent development of agriculture in the Holocene, resulted in waves of linguistic diversification and replacement across the planet. Analogous to the growth of populations or the speciation of biological organisms, languages diversify over time to form phylogenies of language families. However, the dynamics of this diversification process are unclear. Bayesian methods applied to lexical and phonetic data have created dated linguistic phylogenies for 18 language families encompassing ~3,000 of the world’s ~7,000 extant languages. In this paper we use these phylogenies to quantify how fast languages expand and diversify through time both within and across language families. The overall diversification rate of languages in our sample is ~0.001 yr^-1^ (or a doubling time of ~700 yr) over the last 6,000 years with evidence for nonlinear dynamics in language diversification rates over time, where both within and across language families, diversity initially increases rapidly and then slows. The expansion, evolution, and diversification of languages as they spread around the planet was a non-constant process.

## Introduction

As the geographic range of the human species expanded throughout Africa and beyond, eventually including the majority of the world’s terrestrial environments, human populations and their cultures diversified [1]. Today there are over 7,000 languages around the planet [1,2], more than the total number of mammal species [3]. Moreover, most of the world’s current languages belong to agricultural language families [4], and so the majority of current ethnolinguistic diversity has most likely evolved only in the last ten thousand years since the Neolithic [5–7].

While recent research shows that the global biogeographic distribution of linguistic diversity is closely correlated with climatic, environmental, socioeconomic and demographic processes [8–10], the rate at which this diversity evolved and whether it varied over time is unclear [11–14]. Similar questions are asked in the evolutionary dynamics of biodiversity [15]. Under one scenario, rates of linguistic diversification may slow through time as populations compete increasingly for finite resources and space in environments, in which case diversification eventually slows, saturates and asymptotes toward a quasi-equilibrium; this is the negative density-dependent model. Or, perhaps no stable equilibrium exists because between-group competition, or other mechanisms, continuously drive increasing ethnolinguistic diversification and replacement independent of larger environmental constraints; this is a positive density-dependent model where diversity increases diversification rates. Alternatively, ethnolinguistic diversity may be out of equilibrium because of the relatively recent agricultural revolution, and associated population growth that replaced hunter-gatherer ethnolinguistic diversity over large parts of the planet; this is a non-equilibrium model. If this were the case, increasing post-Neolithic population growth toward the present may be driving novel forms of diversification processes not found in hunter-gatherer populations.

**Fig 1:**
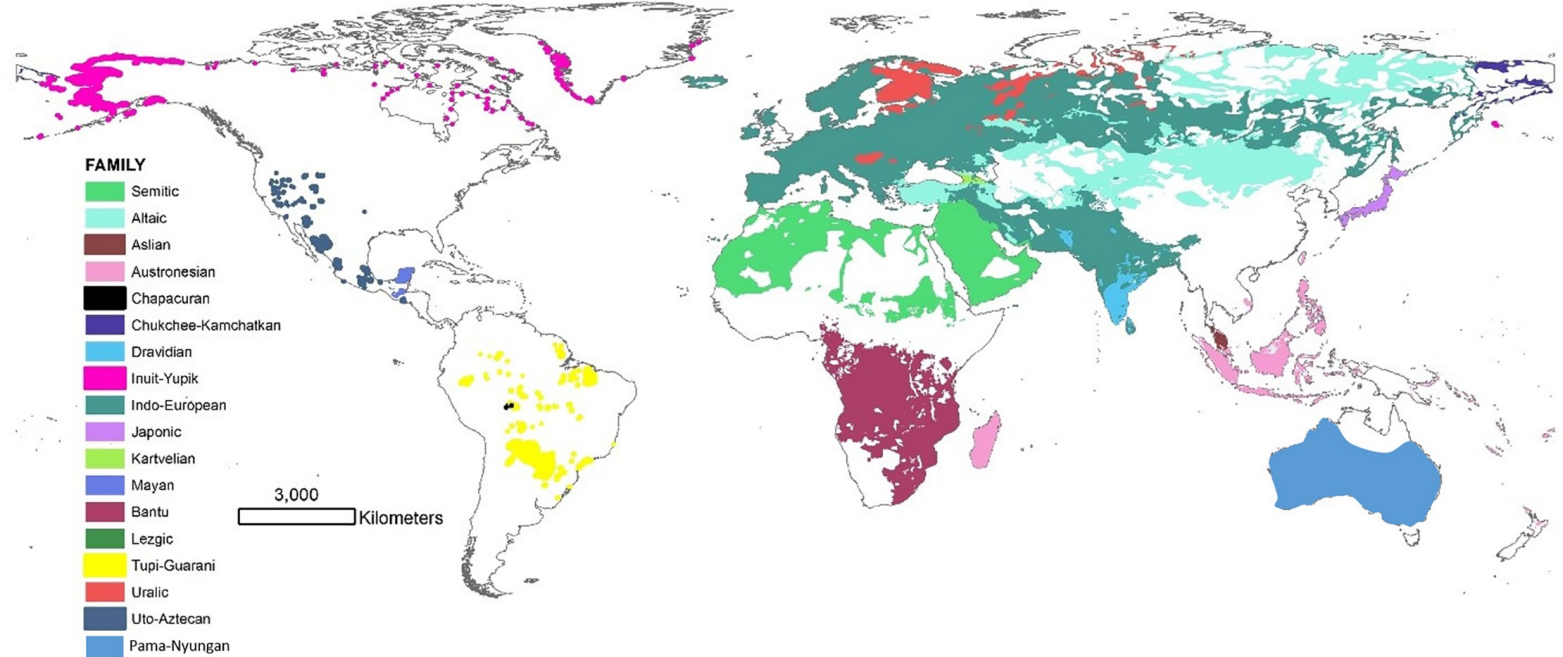
Map of the language families in our sample using polygons from the Ethnologue [2].

To understand how linguistic diversification played out over time we focus on the structure of linguistic phylogenies. Phylogenetic trees based on linguistic variation have proven to be a powerful analytical tool for reconstructing human population and cultural histories [16–18]. By utilizing a Bayesian statistical approach on the systematic codings of linguistic cognates or phonetic data, these methods have generated dated phylogenies for many of the world’s language families, including the large families Austronesian [16], Bantu [17], and Indo-European [18] (Fig 1). These phylogenies allow us to estimate rates of linguistic diversification, show how they vary through time, and compare rates across different language families.

In a simple binary branching process, the number of languages, *N*, will increase exponentially with time, *t*, following the exponential growth function, *N*(*t*) = *N*_0_*e*^*rt*^, where *r* = *b*−*d*, the net difference in language speciations, *b* and extinctions, *d*. Starting from the root of a language family, *N*_0_ = 1, and solving for the net diversification rate, *r*, we define

r≡lnN(t)/t. (1)

**Fig 2:**
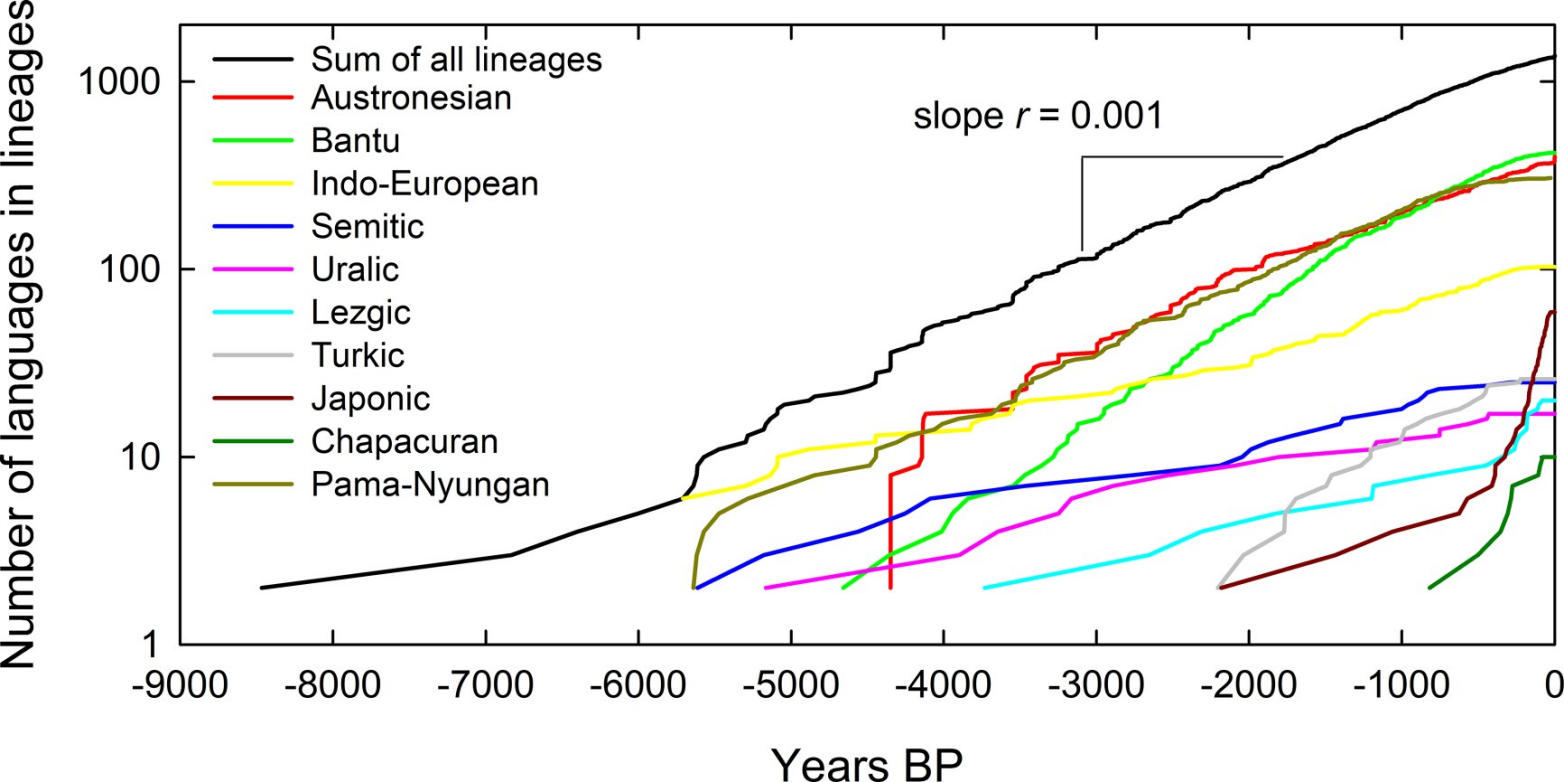
Lineages-through-time plots for 10 language families and the summed total lineages. The number of lineages on the y-axis is on a log scale such that the slopes of these lines are estimates of diversification rate *r*. While there is considerable variation in diversification rates within and across language families, the sum of the lineages suggests an approximately constant diversification rate overall.

If the slope, *r*, of the number of languages, *N*, through time, *t*, is linear in a semi-log plot then the diversification rate is multiplicative and approximately constant, and therefore exponential [19–21] (Fig 2). However, if diversification rates *r* vary through time, either because of time-varying speciation or extinction rates [22, 23], then the slope *r* will not be linear on a semi-log scale. Therefore, *r* is not constant and the diversification rate is time-dependent. To explore the diversification rates we first plot data for all lineages, as well as the sum of all lineages, in Fig 2. Second, for each lineage we then examine the distribution of diversification rates.

The first basic rate of interest for each phylogenetic tree is the overall diversification rate, *r*_*E*_, which is simply the maximum size of the language family (i.e., the number of individual languages), *N*_max_ of the *i*th tree divided by its’ time depth, *T*_*i*_ or

$$r_{E,i}=\ln N_{\max,i}/T_{i}.$$ (2)

From the overall diversification rate we then establish a doubling time,

$$t_{2,i}=\ln2/r_{E,i}$$ (3)

which is the number of years it takes for the number of languages in the *i*th tree to double in size (Table 1).

**Table 1:** Language families analyzed in this study.

Family	N sample	N Ethno	N Glotto	Root age	r_E_ Ethno	Doubling time	r_E_ Glotto	Doubling time	Source
**Kartvelian**		5	6	5500	0.00029	2390	0.00032	2127	[38]
**Dravidian**		85	80	4500	0.00034	2039	0.00034	2056	[73]
**Mayan**		35	33	6500	0.00055	1260	0.00054	1288	[39]
Lezgic*	20	9	10	3730	0.00059	1175	0.00062	1122	[40]
**Inuit-Yupik**		11	12	4000	0.0006	1155	0.00062	1115	[38]
**Uralic**	17	39	48	5300	0.00069	1005	0.00073	948	[41]
Indo-European*	103	449	584	8700	0.0007	990	0.00073	946	[18]
Aslian*		18	19	4000	0.00072	963	0.00074	941	[42]
**Semitic**	25	78	97	5614	0.00078	889	0.00081	850	[43]
**Uto-Aztecan**		61	69	5000	0.00082	845	0.00085	818	[44]
Japonic*	59	12	15	2182	0.00114	608	0.00124	558	[45]
**Bantu**	409	668	721	4800	0.00136	510	0.00137	505	[17]
**Austronesian**	398	1268	1276	5230	0.00137	506	0.00137	506	[16]
Chapacuran*	10	5	12	1039	0.00155	447	0.00239	289	[46]
**Tupi-Guarani**		51	48	2500	0.00157	441	0.00155	447	[47]
**Chukchee-Kamchatkan**		5	5	1000	0.00161	431	0.00161	430	[38]
**Turkic**	26	41	44	2206	0.00168	413	0.00172	404	[48]
**Pama-Nyungan**	306	285	248	5671	0.00099	695	0.00097	712	[74]

In the Family column, “*” indicates that the tree includes both language and dialect data. Sample sizes are given for language numbers in the available phylogenies, including dialects where applicable, and the total number of extant languages within families from both the Ethnologue [1] and the Glottolog [2].

Diversification rates are estimated from Eq 1 using only extant languages (natural-logged) divided by estimated root age in years using both the Ethnologue and Glottolog data.

In evolutionary biology decreasing rates of species diversification through time are often interpreted as evidence for negative density dependence where ecological niches become saturated and limit the opportunities for further speciation [24–29]. Speciation and extinction rates often vary through time leading to expanding or contracting biodiversity and are not well fit by simple exponential growth models [15, 25–27, 30–37]. Here, we ask whether linguistic diversity shows similar evolutionary dynamics. As diversification rates are known to vary within large language families [16–18], we calculate diversification rates between successive time periods,

$$r_{i}\left(t\right)=\Delta \ln N_{i}/\Delta t.$$ (4)

Therefore, for each phylogeny we calculate multiple diversification rates and ask how they change over time, and in response to the number of languages. Because the time depth, *T*_*i*_, varies across the lineages between 1,000 to 13,000 years, and the number of languages within lineages varies from 5 to 1,268, we standardize the time depth and size of each language family, so *N*_*i*_(*t*)' = *N*_*i*_(*t*)/*N*_*i*,max_ and *t*_*i*_' = *t*_*i*_/*T*_*i*_. Therefore, *N*_*i*_(*t*)' is the proportion of the total number of languages at time, *t*, in the *i*th language family and time, *t*_*i*_', is a constant fraction of the time depth (or root age) of the *i*th language family (see S1 and S2 Figs). To calculate the rate of diversification of the trees we then divided the normalized sizes of the lineages in to ten bins of 0.1*t*' each, thus making the number of measureable diversification rates across the lineages directly comparable. Therefore, we are measuring the diversification rate over each consecutive 10% increment in time within each phylogenetic tree, regardless of the actual time depth of the tree thus making the diversification rates directly comparable across trees. All data and code used in the analysis is included in a “README.txt” file attached to this paper (S1 File).

## Results

### Phylogenetic uncertainty

In the following results we do not report phylogenetic uncertainty as the data we used to calculate estimates of diversification rates come from published consensus trees, not the raw data originally used by each study. As such, we cannot model the statistical error associated with the robustness of each tree stemming either from missing data, or lack of information on the relationships between languages. The degree of statistical error likely varies widely across families and through time as sample sizes vary, and the older the phylogeny, the greater the likelihood for multiple sources of error to compound (i.e., incompleteness and uncertainty). Moreover, different language families have been subject to different levels of research intensity by different scholars resulting in unavoidable measurement error across the data set.

### Lineages through time

Lineages-through-time plots visualize the growth of lineages in a phylogeny [20]. Fig 2 shows the 10 phylogenies in our dataset that have topologies (colored lines) and the sum of all lineages across the 10 phylogenies (black line). Fig 2 shows that the diversification rate for the sum of all lineages *r* = 0.001 yr^-1^ (regression: *F*_1,377_ = 229,736.79, *r*^2^ = 99.4%, *p*<0.001). However, Fig 2 also shows that diversification rates vary both within and across language families. Some rates slow with time, others increase, while others remain stable. For example, diversification rates seem to decrease with time within the Austronesian, Pama-Nyugan, and Bantu lineages. Diversification rates in the Lezgic and Japonic lineages seem to increase through time, and while the sum of all lineages combined seems to be relatively constant, there is a slight decrease through time. Table 1 presents the diversification rates and doubling times for all lineages.

### Sum of all lineage diversification rates

**Fig 3:**
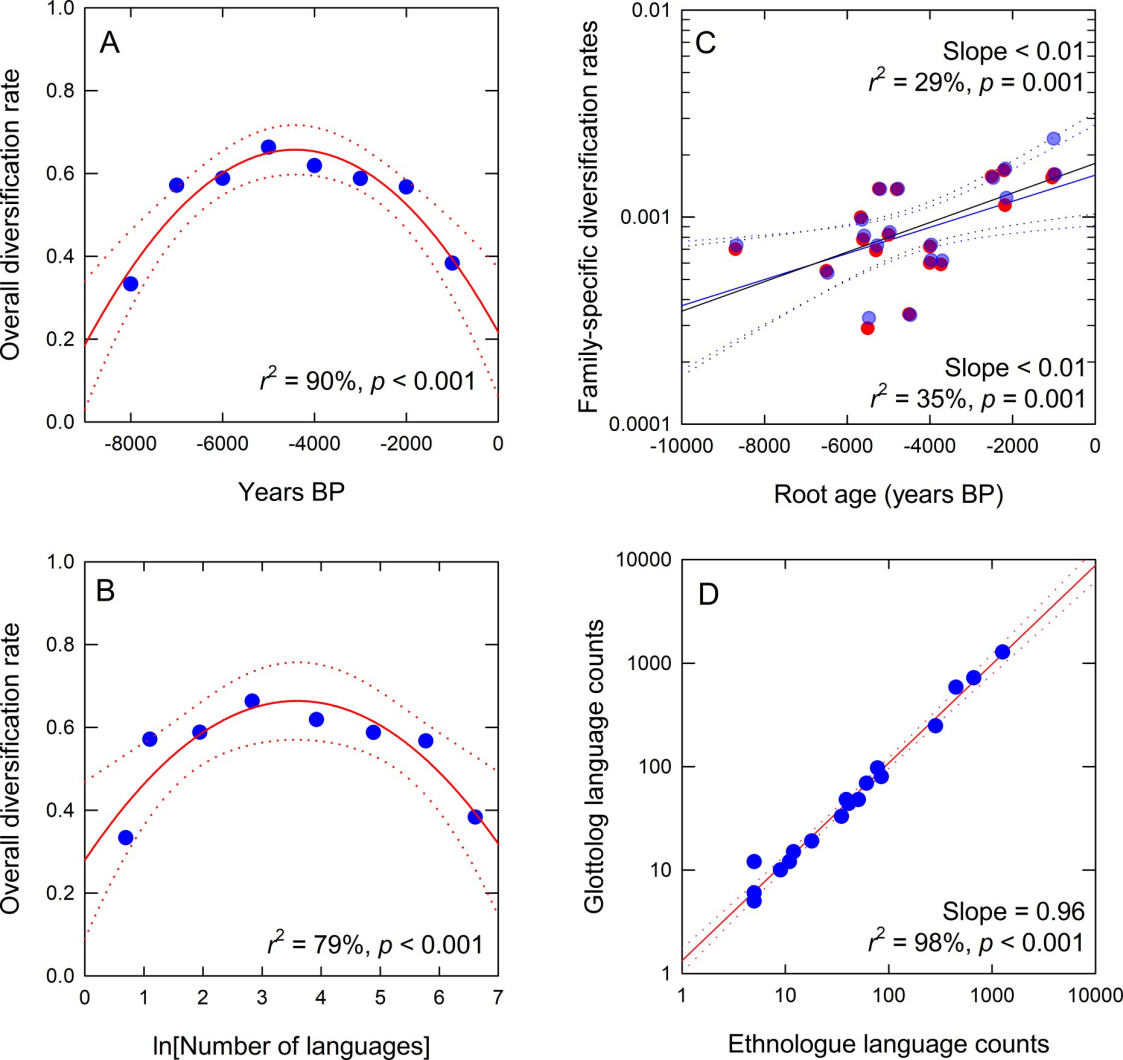
Diversification and diversification rates within and across lineages. A) Overall diversification rates over time fit with a quadratic function (red dotted lines are 95% confidence intervals around the slope). B) Overall diversification rates and the total number of languages, with a quadratic function fit to logged data. C) Diversification rates across lineages by their root ages fit by OLS regression. D) Correlation between the Glottolog and Ethnologue language estimates.

To examine how overall diversification rates change through time we estimate the change in the sum of all lineages per millennia by averaging the data over 1,000 year bins and calculating the change over time. While the black line in Fig 2 is relatively linear on a semi-log scale, Fig 3A shows that the diversification rate changes nonlinearly over time as it is well-fit by a quadratic function (regression: *F*_8_ = 22.69, *r*^2^ = 90%, *p*<0.01). Diversity initially increases (i.e., *b*>*d*) until an inflection ~5,000 BP after which diversity decreases toward the present (i.e., *b*<*d*). Fig 3B shows that the diversification rates are highly nonlinear with the number of languages, as shown by a quadratic function fit to the logged data, therefore capturing the skew (regression: *F*_9_ = 9.45, *r*^2^ = 79%, *p* = 0.02): Diversification rates initially exhibit strong positive density-dependence, where diversification rates increase rapidly with low number of languages, reaching maximum rates at 12 languages, thereafter declining steadily with increasing languages, i.e., negative density-dependence.

### Diversification rates across lineages

Table 1 presents the diversification rates for all 18 lineages for which we have data. The mean rate of diversification across the full sample is 0.001 yr^-1^ (bootstrapped 95% confidence interval = 0.0004–0.001), or a doubling time of ~770 years, similar to the estimated rate from the summed lineages. Using both the Ethnologue and Glottolog data, Fig 3C shows that the diversification rates of phylogenetic trees increases exponentially through time (regression: Ethnologue, *F*_18_ = 12.6, *r*^2^ = 43%, *p* = 0.003; Glottolog, *F*_18_ = 16.4, *r*^2^ = 49%, *p* < 0.001;) by ~20% per millennia, indicating that linguistic diversification is not constant through time. Results do not differ across the data sets, and Fig 3D shows that the data sets are highly correlated.

Linguistic diversification rates across families appear to increase by nearly an order of magnitude through time (Fig 3C). Taken at face value, diversification rates show an exponential increase over time with a slope of 0.001. As such, the doubling time of diversification rates is about 7,000 years. However, this time dependency must be, at least in part, due to an unavoidable “pull of the present” effect [20,21]. This is because language families that arose recently have had less time to undergo extinction than older families. Moreover, our sample does not include many of the world’s smaller families and isolates. We can assume that these slow-growing languages would populate the lower right of Fig 3C to form a wedge shape to the graph and flatten the slope. For these reasons, an overall average diversification rate, as calculated in the previous paragraph, is a good estimate of the rate at which language spread and diversified around the world over the Holocene.

### Diversification rates within lineages

**Fig 4:**
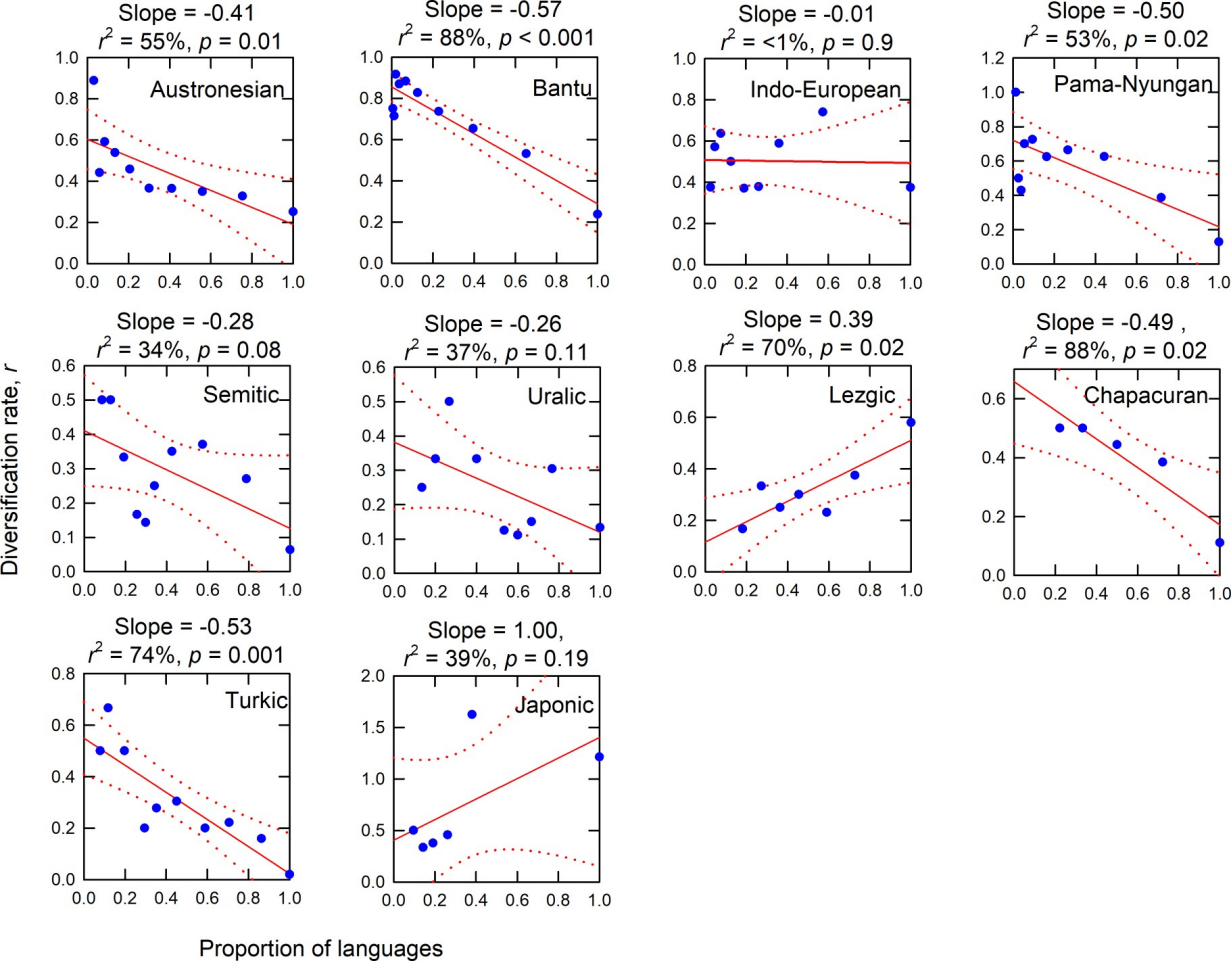
Different forms of density-dependent diversification rates among lineages.

To examine the nature of diversification rates within lineages we recognize three basic forms of potential density-dependence; exponential growth (i.e., constant diversification rates over time), slope = 0; negative density-dependence, slope < 0, where diversification rates slow over time; and positive density-dependence, slope > 0, where diversification rates increase over time. Fig 4 shows the same diversification rates for the nine lineages plotted as a function of the changing number of languages within lineages (statistics of each are given in the title of each panel). Seven of the ten lineages show evidence of negative density-dependence (i.e., slopes > 0): these are the Austronesian, Pama-Nyungan, Bantu, Turkic, Semitic, Uralic, and Chapacuran phylogenies. The Lezgic lineage and Japonic indicate positive density-dependence (slopes > 0). Indo-European exhibits exponential growth independent of density (slope = 0). S2 Fig in the online Supplementary Material show similar results by time.

## Discussion

Our results indicate that rates of linguistic diversification are not constant through time, neither as a whole, nor within most of the individual lineages. Overall rates of diversification across all lineages initially increase for the first few thousand years of the Holocene and thus exhibit positive density-dependence where the actual rate of diversification itself increases with each new language. After the inflection point ~5,000 BP, the diversification rate then decreases, thus exhibiting negative density-dependence, likely reflecting increased competition and saturation of populations on landscapes as languages expand. We find strong evidence for similar patterns within individual lineages where diversification rates are most rapid during the earliest stages of diversification (and at the smallest number of languages), indicating that as new lineages are born, they diversify in punctuated bursts. Lezgic exhibits positive density-dependence, where, similar to the initial stages of overall diversity in the early Holocene, the rate of diversification increases through time toward the present and continues to diversify (i.e., diversity begets diversity). Although the slopes of the Semitic and Uralic are negative, and Japonic is positive, they are not significantly different from 0 and so should be viewed as inconclusive sources of evidence of density-dependent diversification. Indo-European shows no time-dependent trend and so diversification seems to be constant over time. A clear implication of these results is that empirically, the process of language diversification is not well captured by the Yule model, a commonly chosen prior in Bayesian phylogenies, which models diversification as a stochastic, constant branching process [49–52]. However, the extent to which tree structures are robust to the choice of priors is a matter of debate [52,53].

The average diversification rate *r*_*E*_ of 0.001 yr^-1^ over all lineages means that the doubling time of the world’s linguistic diversity is on the order of ~700 years. This diversification is driven by the underlying exponential growth rate of populations as they expanded from their origin [8–11]. When measured ethnographically, growth rates of natural-fertility human populations are often in the range of *r* ~ 2–4% yr^-1^ [54,55]. Human populations therefore have the capacity to grow 20–40 times faster than languages diversify. However, the long-term growth rate of global human populations over the last 6,000 years, up until the industrial revolution, is also on the order of 0.001 yr^-1^ [56–58]. This suggests that language diversification and population growth rate on a global level are closely coupled and at near equilibrium levels (i.e., growth rates are only slightly above zero).

It might be the case that competition for limited resources forces local populations to diverge ethnolinguistically, resulting in some positive density dependence. Like species, linguistic diversification experiences punctuated bursts as languages go through splitting events [59,60]. Rates of new word gain are also faster in larger populations [61,62], although others have argued for faster linguistic evolution in smaller populations [63]. A combination of larger populations and more competition could therefore fuel continuing ethnolinguistic diversification, but it remains an open question as to whether competition alone is sufficient to account for this dynamic. Islands are ideal test cases to establish whether ethnolinguistic diversity continues to increase once equilibrium population sizes have been reached. Indeed, linguistic diversity on islands has been shown to increase with time since initial settlement [61,64].

Language families spread and diversified over the Holocene at nonlinear, density-dependent rates. Like other forms of biodiversity, ethnolinguistic diversity may have continued to expand were it not for recent extinction events associated with globalization [65–68]. Interestingly, the data we present here suggests that the current language extinction crisis is part of a larger process of linguistic replacement that began in the mid-Holocene, ~5,000 years ago with the expansion of agricultural language families, now driven by the expansion of global languages central to the expansion of global trade networks.

## Material and methods

### Linguistic phylogenies

We searched for the keywords “Bayesian linguistic phylogeny” to find studies that use Bayesian phylogenetic methods to attach known calendar dates to nodes of language trees as a means to estimate root ages. We found 19 such linguistic phylogenies (Table 1). Of these we were able to obtain 10 of the actual consensus tree files with the highest posterior probabilities either from online supplementary material or by contacting corresponding authors. Unfortunately, we were not able to obtain error estimates for the consensus trees from the data made available.

Character-based phylogenetic methods using cognate codings of basic vocabulary words or phonetic data are useful for inferring the internal classifications and divergence dates of recent language family expansions. These methods are superior to traditional techniques in several ways by allowing for different rates of change between cognate sets and between different lineages, and by explicitly taking into account available archaeological dates and historical events to infer divergence dates [69]. While alternative phylogenetic methods exist [70–72], there is no competing alternative for our purposes because our analyses require a systematic approach that produces time-dated phylogenies.

The three largest language families in our sample are Austronesian [16], Bantu [17], and Indo-European [18]. Of these Indo-European has proven the most divisive with some arguing for the Kurgan expansion with homelands in the Pontic steppe dating 6,000 years ago versus others supporting the Anatolian expansion of farming around 8,700 years ago [10,18,68]. We opt for the latter following our inclusion criteria of Bayesian methods. Five families in our sample are Eurasiatic language families with estimated dates taken from Pagel and colleagues’ study of ultraconserved words [38]. These families are some of the oldest in our sample, and therefore generally considered the most controversial, but removing them from the sample yields only a slightly faster average diversification rate of 0.00104 yr^-1^. The remaining language families in the sample are more recent expansions (Table 1), hence their divergence dates are likely more accurate.

### Analyses

Lineage-through-time plots were created with the *ape* package in R. The sum of the lineages line in Fig 2 is created by simply aligning the 10 available phylogenies together using absolute time as in the graph and adding all of the lineages together. How exactly each phylogeny relates to others at the basal node is not necessary information for the lineage-through-time plot since each additional lineage is simply counted at each point in time.

For within-family analyses of diversification rates as a function of time, we used the *R* package *TESS* [36]. This is a stochastic-branching model that flexibly fits time-varying speciation and extinction rates and accounts for incomplete sampling [37]. See Supplementary Material for complete details, *R* code, and tree data. The assumption of constant diversification rate *r* is a reasonable assumption for some of these language families so we made this assumption for each family in the full sample of 19. Diversification rates were estimated with Eq 1 using the total (natural-logged) number of languages in a family from the Ethnologue [1] and the Glottolog [2], along with the estimated root age of the tree from the original studies (Table 1). Doubling times were calculated as in Eq 3 [24]. While 4 of the phylogenies include dialects, we calculate diversification with counts of languages only.

## Supporting information

S1 Fig Standardized proportion of languages and phylogenetic age for the language families used in the paper. These are the data standardized by the number of languages in a language family and the longevity of each language family.
(JPG)

S2 Fig Diversification rates as a function of normalized time for each language family. These panels show the diversification rates within each language family over standardized time (i.e., the relative longevity of each language family).
(TIF)

S1 File The data and R code used in the paper. All data and code used in this paper are available in this README.txt file.
(TXT)
