# Supplementary figures and images for: Nonlinear diversification rates of linguistic phylogenies over the Holocene
Source: PLoS One. 2019 Jul 17;14(7):e0213126. doi: 10.1371/journal.pone.0213126 (PMC6636708; doi:10.1371/journal.pone.0213126)

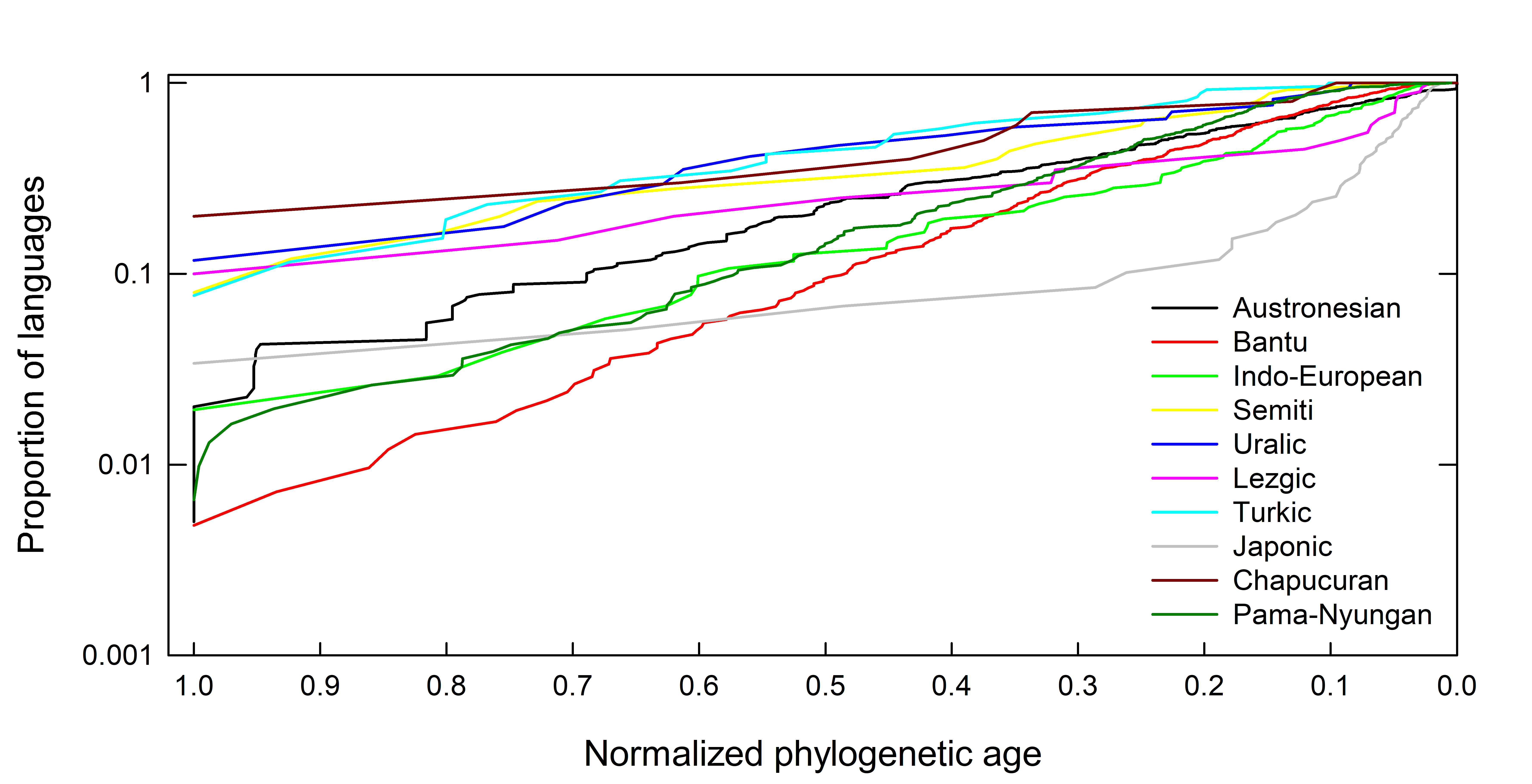

Supplement: S1 Fig — These are the data standardized by the number of languages in a language family and the longevity of each language family. (JPG) [file pone.0213126.s001.JPG]
